# Status of tertiary level online class in Bangladesh: students’ response on preparedness, participation and classroom activities

**DOI:** 10.1016/j.heliyon.2021.e05943

**Published:** 2021-01-15

**Authors:** Md. Al-Amin, Abdullah Al Zubayer, Badhon Deb, Mehedi Hasan

**Affiliations:** a Institute of Education and Research, University of Dhaka, Bangladesh; b Department of Sociology, University of Barishal, Barishal, Bangladesh; c Department of Sociology, Shahjalal University of Science and Technology, Bangladesh; d EQMS Consulting Limited, Bangladesh

**Keywords:** Distance learning, Technological advancement, Online class, Classroom activities, Pandemic

## Abstract

In the era of technology, every time the world confronts any kind of crisis or challenge, we use technology as a weapon. Like other emergencies, as COVID-19 is announced as a pandemic, all countries have started trying to control the situation with technological advancement in the medical sector, educational progress, and in the continuity of productions. As most of the educational institutions have been closed since March and the learning process in higher education has moved online, therefore, developing countries like Bangladesh are also trying to continue classes through the online platform with a lack of technological resources, readiness, and inclusiveness from the perspective of the students. This quantitative study surveyed over 844 students of different universities of Bangladesh to analyze the status of preparedness, participation, and classroom activities through online during the pandemic. The findings revealed a lack of preparedness, participation, and less scope of classroom activities through online learning. Problems of infeasible consistency of the internet and electricity, paying attention, understanding lessons through the online platform are the main constraints of online learning in the developing country. Finding ways of mitigating these problems can be the next subject for further researchers.

## 1 Introduction

The novel coronavirus pandemic is one of the burning issues in the world. The virus has spread rapidly throughout the world although the first human case of COVID-19 was reported in China (Islam et al., 2020). This Pandemic has already engulfed every aspect of our life including education, one of the basic needs of the human being. While online schooling was largely treated as an optional means of education during the prior pandemic, the pandemic period forced it to secure the position of being the central mode of learning. Therefore, online platforms are currently used by educational institutions to support the learning process of students (Mulyanti et al., 2020). According to the World Economic Forum, April 2020, globally, over 1.2 billion children are unable to attend their classes as a result of COVID-19. As a result of this pandemic, schools have been shut down all over the world, and with the advent of distance learning, educational practices have shifted rapidly. Furthermore, several distance learning platforms offer free access to their pandemic resources (Li and Lalani, 2020). But lack of sufficient resources and adequate teacher training, insufficient access to technology, faculty preparedness, late adoption of learners' online learning, lack of technical facilities in rural areas are the most frequently faced obstacles in online education (Dubey and Pandey, 2020).

Almost all the countries are continuing their educational activities online in the pandemic (Mulyanti et al., 2020). In the wake of the coronavirus pandemic, online schooling has opened a new frontier in the Bangladesh education system, recently used as an alternative solution to cover losses in the education field. Online education was familiar before the COVID-19 pandemic in Bangladesh but now it is used intensively to diminish the study gap. As of The Business Standard June 2020, to control the rapid growth of the noxious virus, the Government of Bangladesh had to shut down all the educational and learning institutions from March 17, 2020, after confirming the first COVID-19 patient on March 8, 2020 (Islam et al., 2020).

Subsequently, the Govt. of Bangladesh also declared a special “general leave” from 26 March in the name of “Lockdown”. But among the most socio-economically vulnerable people in our country, got confused between “general leave” and “lockdown”. In a densely populous nation with 165 million people, the so-called lockdown, coronavirus precautions, and social distancing did not operate in the right direction. As a result, Bangladesh is currently witnessing widespread population dissemination, as common citizens are not adequately aware of their health to deter the coronavirus (Shammi et al., 2020). After shutting down all the educational institutions, the Minister of Education instructed all universities to introduce online education. Many public and private universities, colleges, and schools of Bangladesh have moved their classes online. A recent survey has found that although 40% of the students are attending online classes while almost 50% of the students cannot attend the online classes due to a lack of device availability. However, most of them (70%) are from private universities. Students also feel the online courses are somewhat doubtful in terms of viability and efficacy (Islam et al., 2020). Nevertheless, educational institutions are using different mediums of teaching-learning including television, radio, and social media platforms to cover students of different areas yet the household income and expenditure survey 2020 has reported that around 12.70% of the poor families do not have a mobile phone where students need at least a Smartphone and a stable internet connection for attending online education (Tariq and Fami, 2020). The Daily Campus June 2020, has reported that a student and his mother attempted suicide for the student's unwillingness in participating in the online examination (Mamun et al., 2020).

After switching to online classes during the pandemic, students may face new difficulties related to online class preparedness, participation, and activities. Therefore the purpose of this research is to discover the current state of online classes for higher education students.

## 2 Background

During the coronavirus pandemic, online education has certainly been one of the strongest ways of teaching and learning to resolve the academic crisis. In Bangladesh, it has been largely based on the tertiary level of education. It has some advantages, such as keeping students on the right track of learning during crises, allowing students to complete the courses on time, making them confident to undergo online tests, and establishing strong communication between students and teachers. On the contrary, the difficulties of online classes are grappling with the correct adaptability to virtual classrooms, unreliable signals with a high internet price, lack of digital literacy, and maintaining contact with individuals (Alam, 2020). Further, as conventional classes have moved online, most students in developing countries have encountered different problems (Ahmed, 2013). The key obstacles to online education in Bangladesh are the lack of technical resources, high cost, and internet connectivity consistency, the family financial crisis, and the psychological burden on students. The majority of students were also resistant to the proposal to take online classes because of the government's weak technical support (Ramij and Sultana., 2020). A similar finding was perceived to suggest that there was a significant lack of contact between students and teachers in online classes due to two reasons. Firstly, students do not respond during the classroom because of the slow connectivity and high cost of the internet connection. Secondly, most students have forgotten what they learned before leaving their campus. In addition, students suffer from tiredness and loss of attention when taking online courses for a long time (Panday, 2020). Research by Shama and Ikbal. (2020) showed that students were concerned about the study gap and eager to enroll in online classes, but they lack appropriate technical tools, including access to affordable knowledge required for online classes. Faculty members are willing to teach in online classes but universities do not provide faculty members with sufficient technical resources and assistance to conduct successful online classes. Biswas et al. (2020) reported that all developing countries deemed mobile learning to be an effective educational tool, but in Bangladesh, it was not used properly. However, Bangladeshi university students have the willingness to learn online. In addition, they utilize several network protocols and social media for their studies. Students think it is very helpful to recover the study gap during this COVID-19 pandemic time. 40.0% of university students show preference on Facebook for learning, 18.1% on Google, 19.4% on YouTube, 20.0% on Zoom. AlHajri et al. (2017) revealed that the availability of mobile phones among the students is the main reason for shifting their focus to mobile learning to continue their study. Smartphone users now have an increased desire for convenient access to multiple social networking sites. Dutta (2020) concluded that students have used numerous social media apps for the acquisition of academic information. Such as WhatsApp as a messaging application for sharing documents, information & presentations, YouTube for self-learning and Zoom, Skype, Google Meet as video conferencing software is used to accelerate the learning. Apart from the text messengers, video conferencing tools have been widely used for interactions between the teachers and students. Students are now comfortable using technology which helps them to learn, access, share, and create useful information and gain knowledge of a subject.

Rony et al. (2019) noted online teaching-learning has been the way out of a pandemic situation. Yet this new dimension requires instruction for faculty members. In this pandemic case, online teacher training is feasible and can deliver a significant outcome on the change of attitude of faculty members, but there are still some obstacles such as technical hurdles, weak internet access, and an inadequate number of quality trainers. The poor internet connectivity in the marginal areas creates a huge problem, even in the middle classes due to network problems, system errors, software updates, etc. It's useful for nearly 80% of the student participants in the study. Cimermanová (2009) found that e-courses are widely used in the world. The extroverts did not prefer e-learning courses over a face-to-face interaction with teachers. In comparison to independent learners with interpersonal enthusiasm who choose e-learning courses because they obtain similar outcomes in conventional physical courses and online courses. Resistant, reliant, and active students often prefer face-to-face courses. Thus, the form of education does not influence the quality of education. Reeves (2000) claimed that online learning environments have become more prevalent in higher education. Formative and summative assessment, retaining results, and portfolios inside immersive learning settings relative to physical class are the major challenges. In online learning settings, there are three key directions for incorporating alternate appraisal approaches: cognitive, efficiency, portfolio-based evaluations. This required special preparation for university academics, as well as professional support to enhance and incorporate various online activities and assessment programs. Another research, however, has shown that online learning often provides students with many meaningful interactions. Flexibility, cost-effectiveness, resource accessibility, and a well-built class interface, for instance. In addition to the good aspects, the negative perceptions of online learning are time-consuming feedback from instructors, unavailable technical and pedagogical assistance from professors, lack of self-motivation, lack of engagement, unattractive teaching techniques, and course material (Yang & Cornelius, 2004). Although Jaggars et al. (2010) stated that in many cases, students' performance and learning outcomes in online courses are better than those in traditional courses. 70% of online students face technical problems, computer-based learning issues, or other factors related to the online nature of the course. Online courses do not harm prepared and motivated students, and online education offers the benefit of convenience and flexibility in the location and scheduling of their studies. It was seen to be convenient allowing students to study at their own pace and time. Students said that online learning is capable of maintaining a higher level of responsibility and independent learning for their studying. The experiences were not all positive. The inadequate opportunity for human interaction, which was considered necessary to establish peer support and to develop in-depth group discussion on the subject, was a major obstacle to online learning. In this research, students' overall satisfaction with online learning was found to be slightly positive. The focus must be given to teaching and learning to improve the online education system, not merely the technological problem (Sit et al., 2005). A researcher has concluded that online class offers a comparably effective learning alternative. Although student performance is independent of the mode of instruction, certain courses (such as Research Methods) are more challenging to students who persist in the virtual environment than in the classroom. Besides, participation may be less threatening and the quality and quantity of interaction may be increased in online classes. Online communication can be used to improve learning, particularly for students who tend to be introverted in the classroom environment (Ni, 2013). Cherry et al. (2017) observed that distance learning efficacy is not greatly influenced by the classification of teachers, age, years of teaching experience, or the type of organization. Moreover, the number of years of teaching online courses was not associated with faculty satisfaction with teaching online courses or faculty satisfaction with institutional support. Besides, there is a clear beneficial association with technical self-ability in the use of technology-enhanced learning approaches. Obaidullah and Zubayer (2020) revealed that learners are getting used to various online practices especially the growth of soft skills though they face multiple obstacles to participation in e-learning, such as higher prices, broadband latency, network unavailability, and access to computers.

## 3 Methodology

For this research, a cross-sectional study design was used to understand the opinions of students about various aspects of online classes. We collected data from students from different areas through conducting an online survey and also by phone call. The students were selected following the convenience sampling technique which is a non-probabilistic sampling. In this sampling, people are sampled as they are close to hand and can be reached conveniently. We have followed this sampling technique as it was difficult to collect data due to the COVID-19 situation. We have reached 844 students from different areas and universities. 762 of them are currently attending the online classes for their respective courses and 82 of them cannot join due to the lack of some facility issues such as electricity, device, and internet connection. The data analyses have followed a descriptive analysis process, and an independent-sample t-test to reach our research objectives. We have a demographic Table 1 for more information about the sample.

**Table 1:** Demographic information of the sample.

Demographics	Respondents	Percentage (%)
**Gender:**		
Male	490	58%
Female	354	42%
**Residence:**		
City	446	53%
Village	398	47%
**Attending online classes:**		
Yes	762	90%
No	82	10%
**Level of regularity in the online class:**		
Less than 40%:	242	
Between 40%-50%	244	
Between 50%-60%	134	
More than 60%	142	

Table 1 represents the demographic information of the sample where male and city corporation students outnumbered the female and village area students respectively. In this sample, most of the students (762) are attending the online class. But the problem is the regularity of the students on the online platform. 486 students in this study are unable to maintain 50% regularity in the online platform as a result of load shedding, unavailability of necessary devices, an unstable internet connection. The data was collected maintaining ethical issues of research.

## 4 Result and findings

In this section, 14 factors related to the status of students’ preparedness, problems, and activities of the online class have been analyzed according to the survey data. The mean and standard deviation for all 14 factors have been presented in the Table 2. This research followed a nominal scale (Yes/No) to show students' opinions towards different factors. The mean scores were recorded in low, medium, and high band categories for positive responses. In Table 2, the scores were categorically scaled as:

**Table:** 

[0.00–0.49] = low number of positive responses
[0.50–0.74] = moderate number of positive responses
[0.75–1.00] = high number of positive responses

**Table 2:** Students response to different points/components of online class.

Factors	Mean	Standard Deviation
Electricity at student's residence	.97	.166
Having an internet connection	.75	.431
Having a separate reading room	.46	.499
Having a computer	.50	.501
Having an android phone	.93	.261
Electricity during online class time	.51	.493
Unstable internet connection during class time	.75	.407
Paying proper attention during the online class	.33	.462
Regularity of online class	.55	.498
Understanding lesson through the online class	.23	.421
Scope of questioning & answering through online	.82	.384
Possibility of assessments through the distance learning process	.22	.416
Submitting assignment through current distance learning	.38	.487
Effectiveness of online class for the teaching-learning process	.51	.500

The aforementioned Table 2 depicts students' responses on preparedness, problems, and activities for the online class. According to the Table 2, it is crystal clear that students' responses for preparedness related factors were positive except for having a separate reading room. The mean for a separate reading room is below .50 that indicates lesser students have a reading room at their house. So, they do not get a good environment for performing online classes. This table also offers a more positive view in terms of having an internet connection (m = 0.75) android (m = 0.93) and electricity (m = 0.97). So, almost all the students have electricity and an android phone for joining an online class. But the mean for having a computer is 0.50, which is categorized moderately. However, the mean for electricity during the online class (m = 0.51) is not that high. Besides, they face the problem of unstable internet connection (m = 0.75) during class time. It is one of the reasons for students’ irregularity (m = 0.55) in the online class. Students cannot pay proper attention (m = 0.33) to their lesson and they claimed that they did not understand (me = 0.23) the lesson properly online. Moreover, according to the table, the possibility of conducting a formal assessment online is very low (m = 0.22). The submission of the assignment has a mean of .38 which is quite low as well. On the other hand, students can respond by questioning and answering (m = 0.82) during an online class. The mean for this factor is significant. The standard deviation for 12 factors is above 0.38 which means the responses are much scattered. For the rest of the 2 factors, (having electricity and android phone) students have responded quite similarly.

Here in the below section, we can find students' responses to different components of this study.

### 4.1 Students’ preparation for the online platform

**Figure 1:**
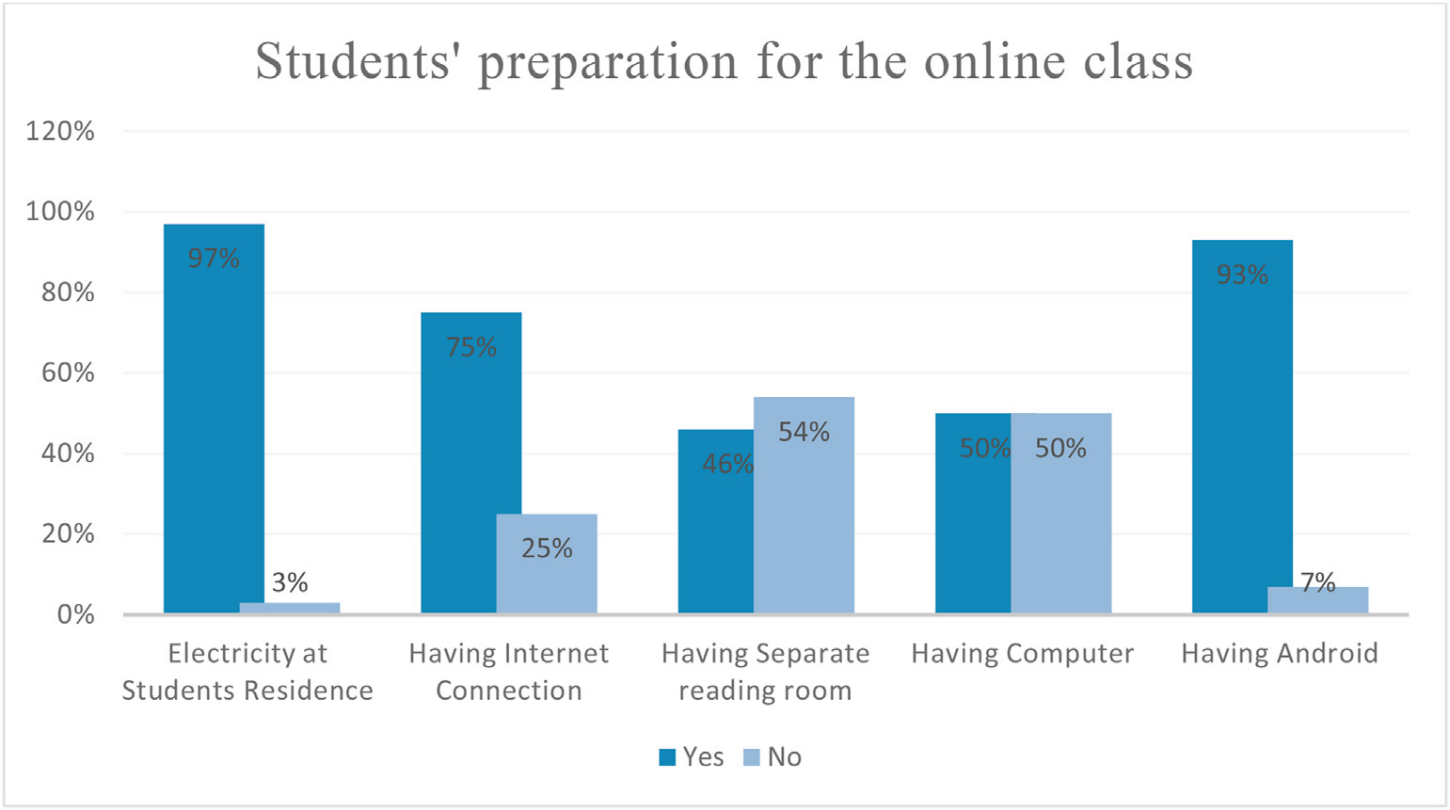
Students' preparation for the online class.

The below Figure 1 shows students’ responses to the preparedness for attending an online class. To be regarded as well prepared, students must have a device, electricity, an internet connection, and a better home environment.

Figure 1 indicates students’ preparation for joining an online class. From Figure 1, it is quite noticeable that most of the students (97%) have electricity in their house as well as an android phone (93%) for joining an online class. However, many students do not have a computer (50%) and an internet connection (25%) which generate the constraints of performing online classes for all. Moreover, the majority of the students (54%) do not have a separate reading room which is also necessary for paying attention in class time.

### 4.2 Problems during online class

**Figure 2:**
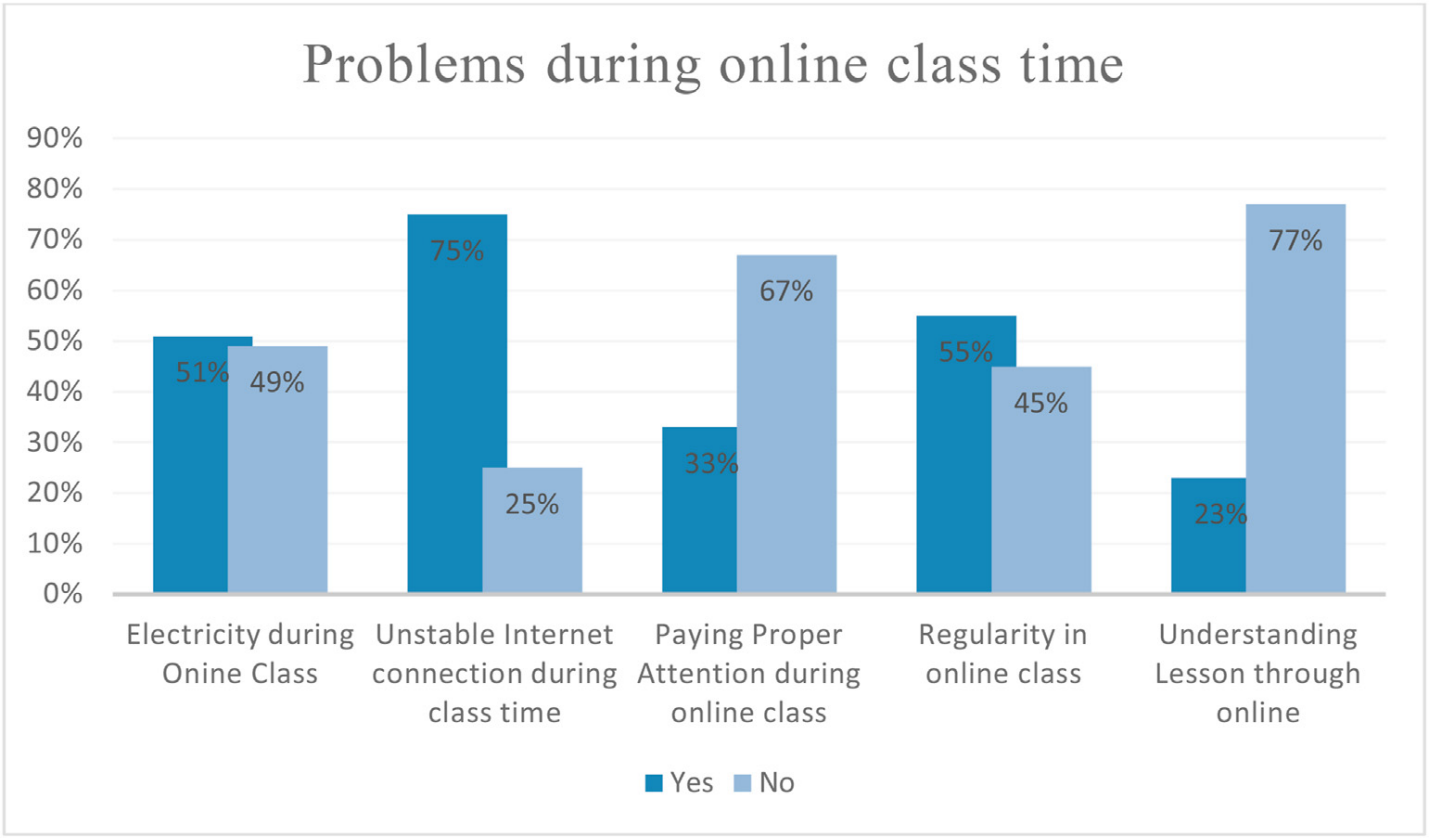
Problems during online class time.

Students face many problems during an online class. According to students, electricity problems or load shedding, inconsistent internet connection, and not understanding topics online have a serious impact on the students' attention. Inconsistent electricity and the internet are major reasons for students’ irregularity in joining the online class. The below graph in Figure 2 shows the percentage of responses for different problems during an online class.

Figure 2 demonstrates that 49% of the students face electricity problems while 75% of students don't have stable internet connection during class time. In terms of paying attention, the majority (67%) responded negatively. Moreover, the response for regularity in attending the online class was moderate as 45% of students responded that they could not join the online platform regularly. They have mentioned unstable internet connection and electricity as constraints for joining online classes. Moreover, only 23% of the students agreed about understanding lessons properly through online classes.

### 4.3 Status of online classroom activities

**Figure 3:**
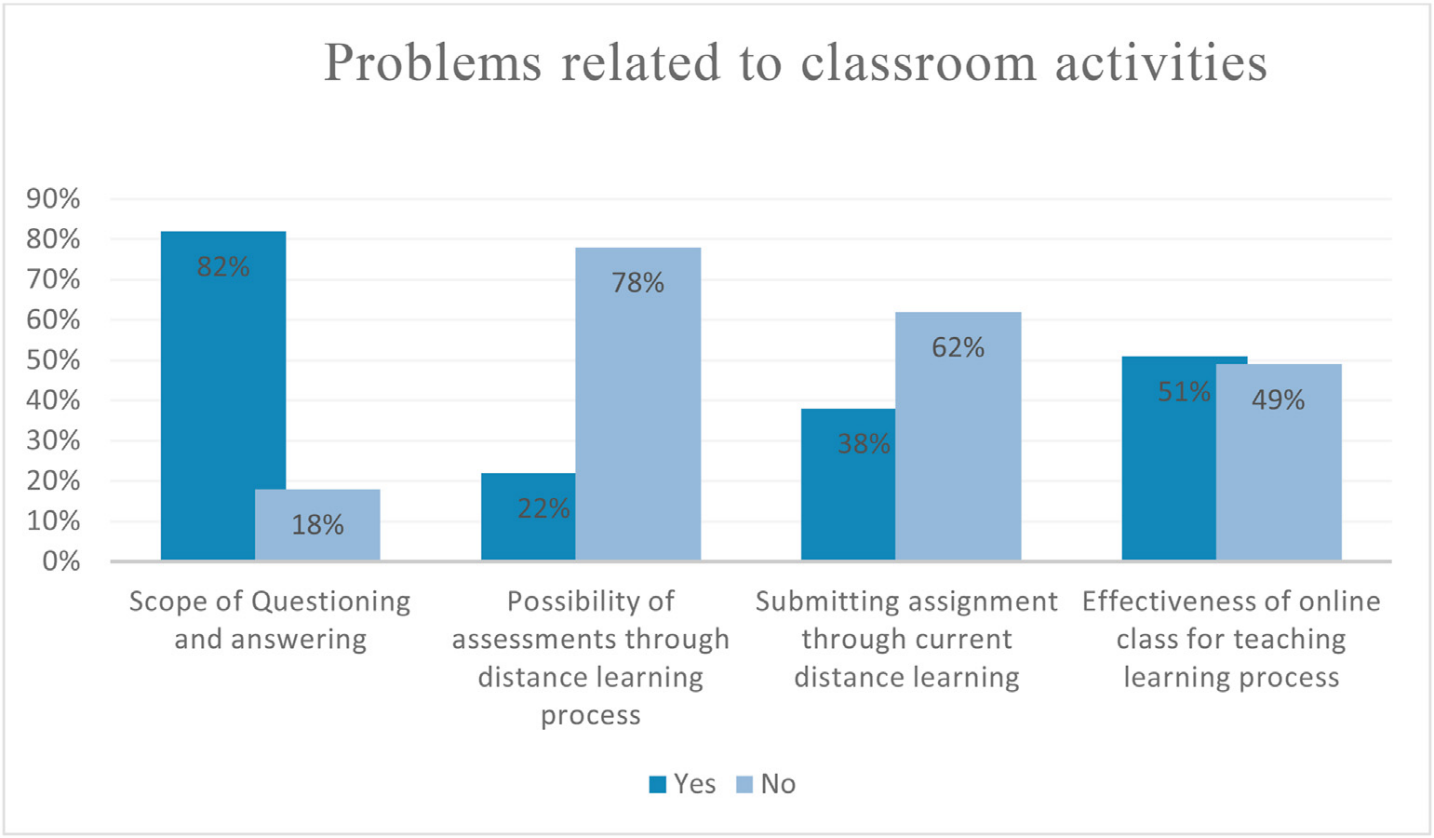
Status of online classroom activities.

The teaching-learning process is a mixture of a lot of activities. It is not only about giving lectures to the audiences but also discussing, responding, assessing the students. Conducting all these activities during online classes are quite difficult. Here in Figure 3, we can find students’ responses to classroom activities.

The graph in Figure 3 shows students get a good scope of questioning and answering during the class though there remains less scope for assessments. 82% of the students positively responded to the scope of questioning and answering but only 22% found online assessment feasible. Similarly, 38% of students responded yes to the feasibility of submitting assignments online. Mixed responses were found for the effectiveness of the online classes in this study. 51% perceived online class effective while their counterparts marked it as ineffective.

### 4.4 Group statistics: comparison of city and village areas students regarding online class

Table 3 shows that there is a significant difference between the respondents from the city and village for some factors such as regularity of online class, paying proper attention during online class, having a computer, having an internet connection, having a separate reading room, unstable internet connection during class time, electricity during online class time. For these factors, the city corporation students are getting more benefits from the online classes than the students from villages.

**Table 3:** Area wise comparison.

Factor	Residence	Mean	Std. Deviation	T-value	Sig. (2 tailed)
Electricity at student residence∗	City Village	.99 .95	.094 .219	2.462	.014
Having an internet connection∗	City Village	.87 .63	.342 .485	5.749	.000
Having a separate reading room∗	City Village	.54 .38	.500 .487	3.249	.001
Having a computer∗	City Village	.63 .35	.484 .477	5.997	.000
Having an android phone	City Village	.94 .91	.235 .288	1.249	.212
Electricity during online class time∗	City Village	.58 .44	.495 .498	2.507	.013
Unstable internet connection during class time∗	City Village	.62 .88	.486 .329	5.279	.000
Paying proper attention during online class∗	City Village	.42 .24	.496 .428	3.474	.001
The regularity of online class∗	City Village	.61 .48	.489 .501	2.578	.010
Understanding lesson through online	City Village	.24 .22	.425 .418	.257	.798
Scope of questioning & Answering through online	City Village	.83 .80	.373 .398	.742	.459
Possibility of assessment through the distance learning process	City Village	.22 .22	.415 .417	-.078	.938
Submitting assignment through current distance learning	City Village	.39 .38	.489 .487	.157	.876
Effectiveness of online class for the teaching-learning process	City Village	.52 .50	.501 .501	.362	.718

*Source*: survey data, 2020

N.B.: 5% level of significance and two-tailed test.

∗ Marked factors have a statistically significant difference for the students of the city and village.

## 5 Discussion

The study has tried to identify different aspects of online classes in Bangladesh. The findings of the research show that students are prepared in terms of a device to join online learning. Another study found similar findings that most of the university students of Bangladesh have a mobile phone to join the online platform (AlHajri et al., 2017). However, this study found that a stable internet connection is a crying need for students. Besides, many students do not have a stable internet connection. Tariq and Fami (2020) have opined in a similar manner and also remarked that a stable internet connection is important for joining the online class. Moreover, our study has found that students do not have a separate reading room which distracts concentration in online classes. The findings of this research also show the irregularity of the students in the online class. The unstable internet connection and electricity are the two main reasons behind the irregularity problem. Students' attendance range was 40%–60% for this study. Another research found that almost 50% are not able to participate in online classes due to the internet and device-related problems (Islam et al., 2020). Moreover, students do not pay attention to the research classes. Research by Panday (2020) mentioned that students suffered from a lack of attention during the online class. students also stated that they did not understand the lesson properly through this new learning platform. The effectiveness of classroom activities also questionable through online platforms which supports the study of Islam et al. (2020). We have found the assessment process as a difficult task in the online class. The research of Reeves (2000) supports our findings of the assessment. It mentioned that formative and summative assessment are the major challenges of online learning. Apart from that, Yang and Cornelius (2004) oppose our finding of questioning & answering during online classes. Students of our study claimed that they could submit queries to their teachers and find answers as well. However, students’ responses oppose the possibility of submitting assignments online.

## 6 Conclusion

The purpose of this study is to understand the current status of students’ preparedness, participation, and classroom activities regarding online class during the pandemic situation. The results show that Bangladesh students have an average level of preparedness for online classes as there are certain limitations related to attendance and activities in the classroom. In terms of multiple factors in the online classroom, a major disparity between rural and urban students is also revealed. So, administration and policymakers should make moves after examining the readiness of the students to ensure inclusiveness in education. For future studies, it will be recommended that further research can be conducted in several developing countries to generalize the findings of this research. More variables can be added to the study and the study can be conducted focusing on primary and secondary level students. Moreover, a qualitative study of students and teachers can be done to make the results more detailed and clarified.

## Declarations

### Author contribution statement

A. Al Zubayer: Conceived and designed the experiments; Performed the experiments; Contributed reagents, materials, analysis tools or data.

Md. Al-Amin: Conceived and designed the experiments; Analyzed and interpreted the data; Contributed reagents, materials, analysis tools or data; Wrote the paper.

M. Hasan: Conceived and designed the experiments, Performed the experiments, Contributed reagents, materials, analysis tools or data.

B. Deb: Conceived and designed the experiments; Performed the experiments; Contributed reagents, materials, analysis tools or data.

### Funding statement

This research did not receive any specific grant from funding agencies in the public, commercial, or not-for-profit sectors.

### Data availability statement

Data will be made available on request.

### Declaration of interests statement

The authors declare no conflict of interest.

### Additional information

No additional information is available for this paper.
